# Postpartum Immune Reconstitution Inflammatory Syndrome Presenting as Massive Bilateral Pleural Effusion in a Previously Healthy Woman: A Case Report

**DOI:** 10.7759/cureus.89565

**Published:** 2025-08-07

**Authors:** Jay Mehta, Nila Mehta

**Affiliations:** 1 Internal Medicine, BayCare Health/Mease Countryside Hospital, Safety Harbor, USA; 2 Obstetrics and Gynecology, Maa N Baby Hospital, Surat, IND

**Keywords:** case report, cesarean section, immune rebound, immune reconstitution inflammatory syndrome, iris, pleural effusion, postpartum

## Abstract

Immune reconstitution inflammatory syndrome (IRIS) is commonly described in individuals recovering from immunosuppression, particularly in HIV-positive patients initiating antiretroviral therapy. However, a similar rebound phenomenon can occur postpartum, a period marked by a shift from an immunotolerant to a pro-inflammatory state. IRIS in this context is underrecognized and may present atypically, complicating timely diagnosis. We report the case of a 35-year-old South Asian woman (G2P2), previously healthy, who developed severe respiratory symptoms within 48 hours postpartum following a planned cesarean section. Despite unremarkable perinatal screening and a smooth surgical course, she experienced an acute onset of breathlessness and dry cough. Imaging revealed massive bilateral pleural effusions with partial lung collapse. Extensive workup ruled out common infectious, thromboembolic, cardiac, and autoimmune etiologies. A therapeutic thoracentesis and empirical treatment with corticosteroids and broad-spectrum antimicrobials led to rapid improvement. Based on the clinical course and exclusion of alternative diagnoses, a diagnosis of postpartum IRIS was considered. This case underscores the importance of considering IRIS in the differential diagnosis of unexplained systemic inflammatory responses in postpartum patients. The postpartum period represents a vulnerable window due to the abrupt immune reconstitution that occurs after childbirth. Early recognition of this entity is crucial for effective management and for preventing unnecessary invasive interventions.

## Introduction

Immune reconstitution inflammatory syndrome (IRIS) describes a paradoxical worsening of pre-existing infections or the unmasking of previously occult infections following recovery of the immune system. Although most well documented in HIV patients starting antiretroviral therapy (ART), IRIS has also been described postpartum, during which the immune system transitions from a tolerogenic to a more inflammatory state [1,2]. This shift, driven by hormonal changes and restoration of cell-mediated immunity, can trigger exaggerated inflammatory responses to subclinical infections or residual antigens [3,4].

Postpartum IRIS is rarely reported but is clinically significant. Recognition is challenging due to its variable presentation and overlap with common obstetric complications.

The case presented herein highlights a severe manifestation of presumed IRIS in the immediate postpartum period, a clinical scenario with limited data currently available in the literature, and underscores the need for heightened clinical vigilance. By contributing to the sparse body of knowledge on postpartum IRIS, this report may aid clinicians in recognizing similar presentations and serve as a reference point for future research.

## Case presentation

A 35-year-old South Asian woman, gravida 2 para 2, with no known comorbidities, was admitted for an elective lower-segment cesarean section (LSCS) at 38 weeks of gestation due to a previous cesarean scar with localized pain and thinning. Routine prenatal infection screenings, including HIV, hepatitis B virus (HBV), hepatitis C virus (HCV), syphilis, and herpes simplex virus (HSV), were negative. Apart from recurrent vulvovaginal candidiasis during pregnancy, her antenatal course was uneventful.

She underwent an uncomplicated LSCS under spinal anesthesia on March 27, 2025, delivering a healthy infant. Prophylactic perioperative medications included intravenous amoxicillin-clavulanate, pantoprazole, ondansetron, tranexamic acid, and metoclopramide. Postoperatively, she received standard uterotonics (carbetocin, methylergometrine, misoprostol), antibiotics (amoxicillin-clavulanate, metronidazole), and supportive care.

On the evening of March 28, 2025 (postoperative day 2), she developed an acute dry cough, dyspnea, orthopnea, and diaphoresis. Oxygen saturation dropped to 92% when supine but improved to 96% when upright. She was afebrile but tachycardic. The initial workup, including an ECG and blood glucose test, was unremarkable. Given concern for cardiopulmonary pathology, she received supplemental oxygen, empiric diuretics, corticosteroids (deflazacort), and broad-spectrum antibiotics (azithromycin and doxycycline).

**Figure 1:**
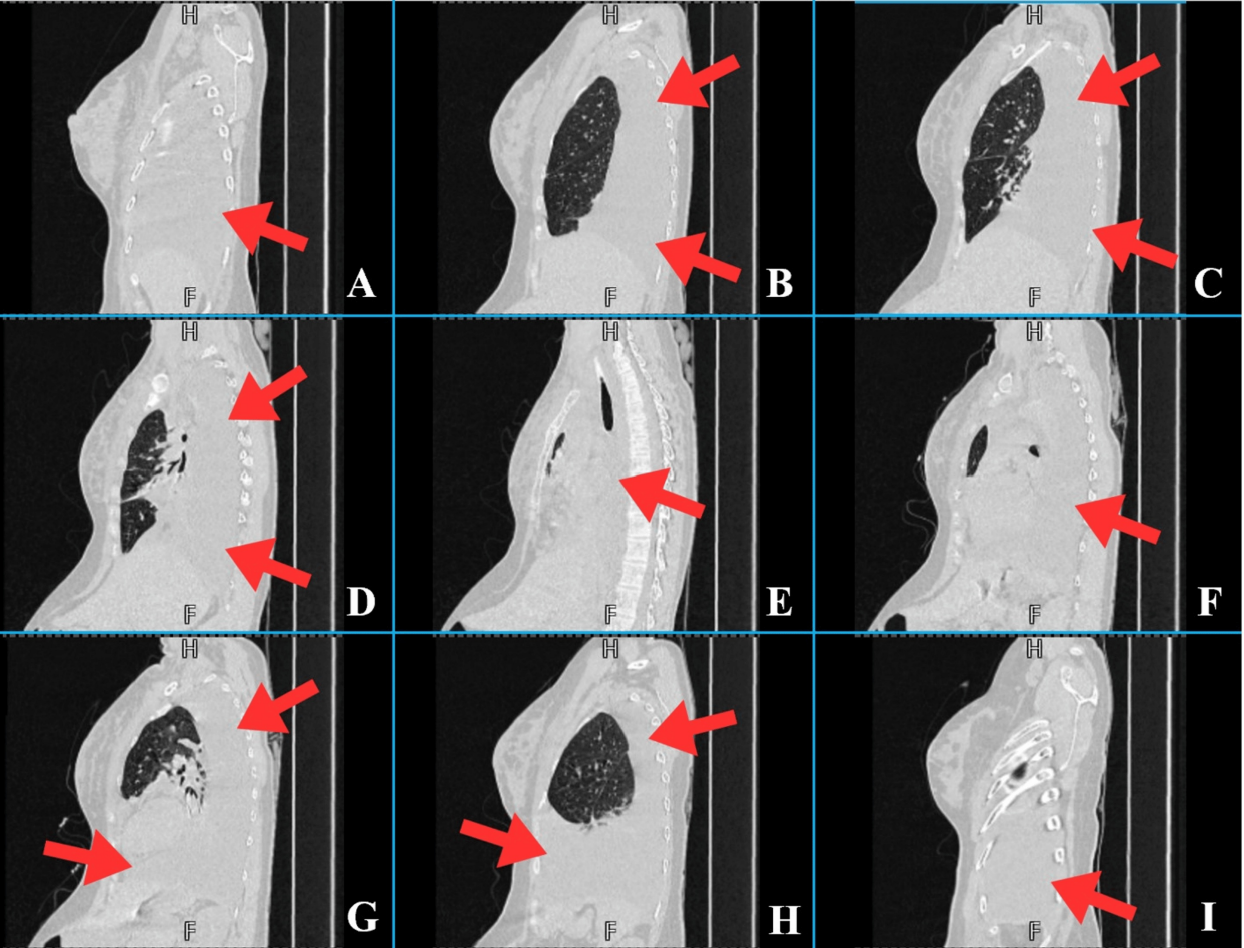
Sequential sagittal high-resolution CT scans of the chest (panels A–I) illustrating findings from the right to the left thoracic cavities (March 29, 2025). A and B: Moderate right-sided pleural effusion with passive collapse of the posterior segment of the right upper lobe (red arrows). C: Right-sided pleural effusion (red arrows) with prominent pulmonary vasculature and passive collapse of the right lung. D and E: Transition across the mediastinum toward the left hemithorax, with visible left-sided pleural effusion (red arrows). F: Moderate-to-large left-sided pleural effusion (red arrow). G: Left-sided pleural effusion (red arrows) accompanied by prominent pulmonary vasculature and passive collapse of the left lung. H and I: Left-sided pleural effusion causing passive collapse of the lower lobe and the posterior segment of the upper lobe of the left lung (red arrows).

**Figure 2:**
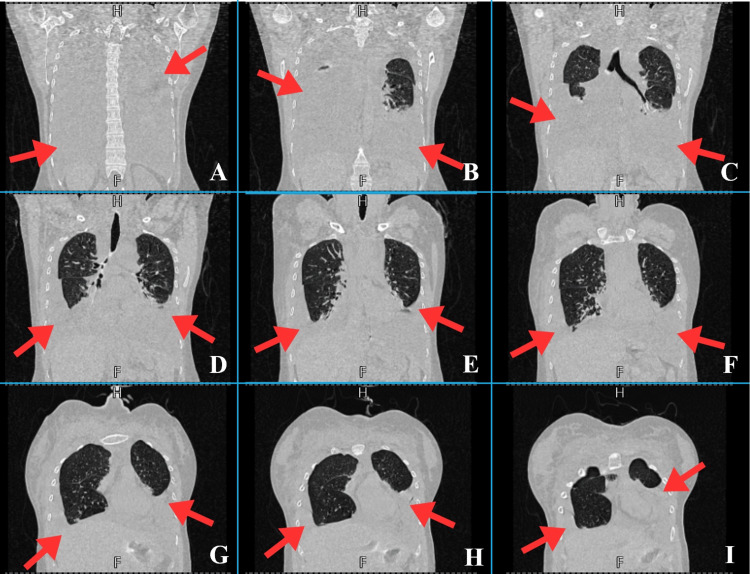
Sequential coronal high-resolution CT scans of the chest (panels A–I) exhibiting thoracic findings at three anatomical levels: posterior, middle, and anterior mediastinum (March 29, 2025). A and B: The posterior mediastinum shows a large bilateral pleural effusion (red arrows). C and D: The trachea and its bifurcation are visible, with moderate-to-large left-sided pleural effusion and moderate right-sided pleural effusion (red arrows), resulting in compression of the basal lung segments. E, F, and G: The middle mediastinum shows bilateral pleural effusions (red arrows) and increased pulmonary vascular markings, consistent with vascular congestion. H and I: The anterior mediastinum shows bilateral pleural effusion, resulting in passive atelectasis of the lower lobes of the lungs (red arrows).

**Figure 3:**
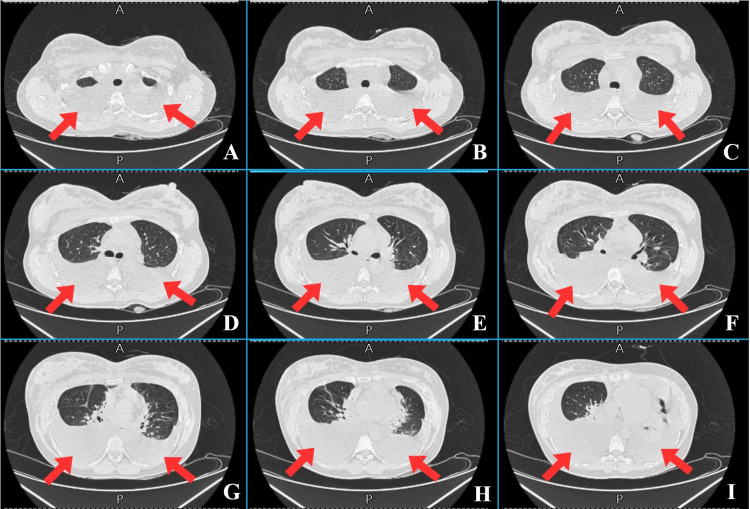
Sequential axial high-resolution CT scans of the chest (panels A–I) demonstrating thoracic findings at three anatomical levels: upper, middle, and lower thoracic cavities (March 29, 2025). A, B, and C: At the upper thoracic level, the images show the trachea and bilateral pleural effusions (red arrows), resulting in passive collapse and atelectasis of the upper lobes of both lungs. D, E, and F: At the mid-thoracic level, the images show the tracheal bifurcation along with moderate bilateral pleural effusion (red arrows). Additionally, there is prominent pulmonary vasculature and partial collapse of the posterior lung segments. G, H, and I: At the lower thoracic level, the images show the heart and its chambers, along with bilateral pleural effusion causing passive collapse of the lower lung lobes (red arrows).

She was transferred to a tertiary care hospital, where an examination revealed reduced breath sounds in both lungs. Investigations included an elevated D-dimer level of 2.09 mg/L FEU (fibrinogen equivalent units) (normal range: <0.50 mg/L), arterial blood gases indicating compensated respiratory alkalosis, echocardiography demonstrating a preserved ejection fraction with no valvular or structural cardiac abnormalities, and a high-resolution CT scan of the chest revealing significant bilateral pleural effusion, dependent lung collapse, and mild pericardial effusion (see Figures 1-3).

Diagnostic thoracentesis yielded 2400 mL of exudative pleural fluid. Pleural fluid analysis results are shown in Table 1.

**Table 1:** Pleural fluid analysis Pleural fluid analysis shows elevated LDH and protein levels with lymphocyte predominance, indicating an underlying inflammatory reaction. In contrast, elevated glucose, negative AFB and Gram stain, and ADA and cell counts within normal limits rule out an infective etiology.

Test	Measured	Normal Value
Lactate dehydrogenase (LDH)	387.3 IU/L	≤200 IU/L
Protein	3.2 g/dL	<2 g/dL
Glucose	163 mg/dL	≥60 mg/dL
Adenosine deaminase (ADA)	16.3 U/L	<40 U/L
Cell count	300 cells/μL (80% lymphocytes)	<1000 cells/µL
Acid-fast bacilli (AFB) and Gram stains	Negative	Negative

Serum inflammatory markers were elevated (CRP: 65 → 29.5 mg/dL) (normal: <10 mg/L), but cultures and viral PCRs, including those for SARS-CoV-2 and influenza strains, were negative. NT-proBNP and amylase/lipase levels were within normal limits. Given low clinical suspicion, autoimmune markers were not tested.

**Figure 4:**
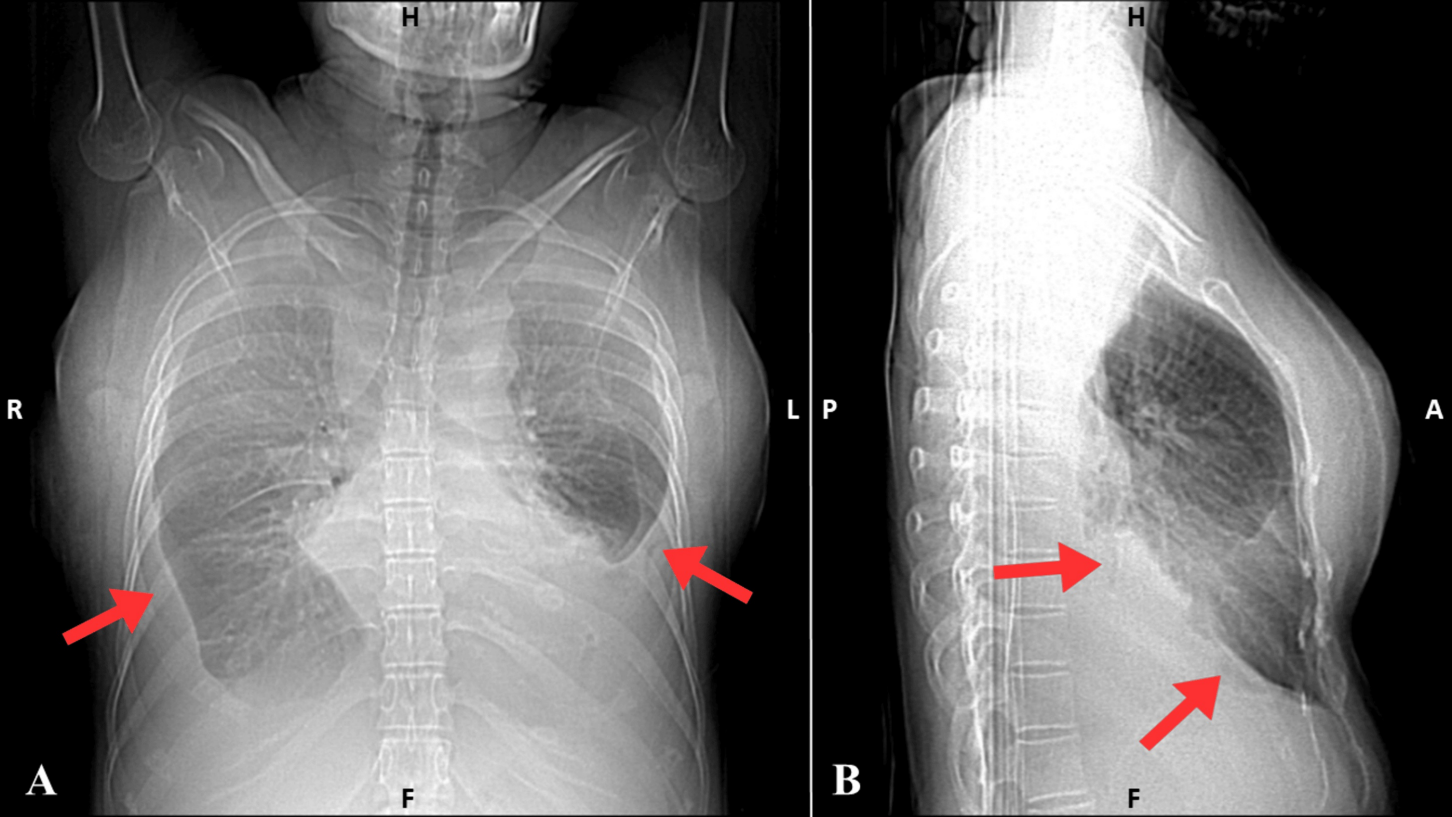
Chest X-ray post-thoracentesis (panels A and B) showing residual pleural effusion (March 30, 2025). A: Anteroposterior (AP) view showing bilateral residual pleural effusions (red arrows) with partial re-expansion of the lungs. B: Lateral view showing residual pleural effusion (red arrows) post-thoracentesis.

Her clinical condition improved with continued corticosteroids and antibiotics (cefoperazone-sulbactam, clindamycin, azithromycin). Repeat chest imaging showed resolving effusions (see Figure 4).

She was discharged on April 1, 2025, with oral antibiotics and a tapering course of corticosteroids. At follow-up on May 6, 2025, she reported complete resolution of her symptoms.

Given the exclusion of infections, cardiac dysfunction, thromboembolic events, and autoimmune causes, a presumptive diagnosis of postpartum IRIS was made.

## Discussion

This case highlights an unusual but instructive instance of IRIS occurring in the immediate postpartum period. While most IRIS literature centers on HIV/AIDS patients commencing ART, similar immunological phenomena may arise after childbirth due to the rapid restoration of immune surveillance [1,5,6].

Peripartum pleural effusion is rare and typically benign [7,8]. Infectious or autoimmune pleuritis, pulmonary embolism, or cardiac failure must be excluded. Our patient underwent a thorough diagnostic workup, including high-resolution computed tomography (HRCT), pleural fluid analysis, and echocardiography, which excluded typical etiologies. Similar presentations have been reported in the absence of an identifiable pathogen, raising suspicion of postpartum IRIS [6,9].

Thapa and Shrestha [1] describe IRIS as a dysregulated immune response that targets latent infections or residual antigens. The postpartum state is recognized as a highly immunologically dynamic period. During pregnancy, immunosuppression mediated by elevated progesterone, cortisol, and IL-10 protects the fetus [2,3]. After delivery, a rapid decline in these hormones restores Th1-type responses, leading to an increase in pro-inflammatory cytokines such as TNF-α and IL-12 [10]. This rebound may unmask subclinical infections or trigger exaggerated inflammatory responses, as in this case.

Importantly, IRIS remains a diagnosis of exclusion, particularly in non-HIV populations, where clinical manifestations are more heterogeneous and often lack the well-characterized trajectories seen in HIV-associated IRIS [1,11,12]. In the postpartum setting specifically, no formal diagnostic criteria have been established, and diagnosis relies on the exclusion of alternative causes along with clinical recognition of an exaggerated inflammatory response to latent or previously treated infections [6,12].

The severity of presentation, rapid fluid accumulation, and dramatic response to immunomodulation distinguish this case. It contributes to the limited pool of literature, reinforcing that IRIS should be considered in the differential diagnosis for postpartum systemic inflammatory presentations, particularly when classical causes are ruled out.

The patient experienced considerable distress from the unexpected respiratory symptoms following what she thought would be a routine surgical delivery. However, she felt relieved once her symptoms resolved and expressed gratitude for the multidisciplinary care she received. She was able to return to breastfeeding and caring for her infant without any issues.

## Conclusions

Postpartum IRIS, though uncommon, is a critical consideration in patients presenting with acute inflammatory syndromes shortly after delivery. Recognition of this condition requires a high level of clinical suspicion, particularly when other causes have been ruled out. Timely intervention with immunosuppressive therapy can lead to rapid clinical improvement. Increased awareness of this phenomenon may prevent misdiagnosis and unnecessary invasive procedures.
